# 2-Phenyl­imidazolium hemi(benzene-1,4-dicarboxyl­ate) trihydrate

**DOI:** 10.1107/S1600536811044953

**Published:** 2011-11-02

**Authors:** Heng-Da Li

**Affiliations:** a Department of Chemistry, Jilin Normal University, Siping 136000, People’s Republic of China

## Abstract

The asymmetric unit of the title compound, C_9_H_9_N_2_^+.^0.5C_8_H_4_O_4_^−^·3H_2_O, contains one 2-phenyl­imidazolium cation, half a benzene-1,4-dicarboxyl­ate anion and three water mol­ecules, which are connected by O—H⋯O and N—H⋯O hydrogen bonds into a three-dimensional network.

## Related literature

For the structures of 2-phenyl­imidazolium nitrate and 2-phenyl­imidazolium acetate, see: Xia *et al.* (2009 ▶); Xia & Yao (2010 ▶).
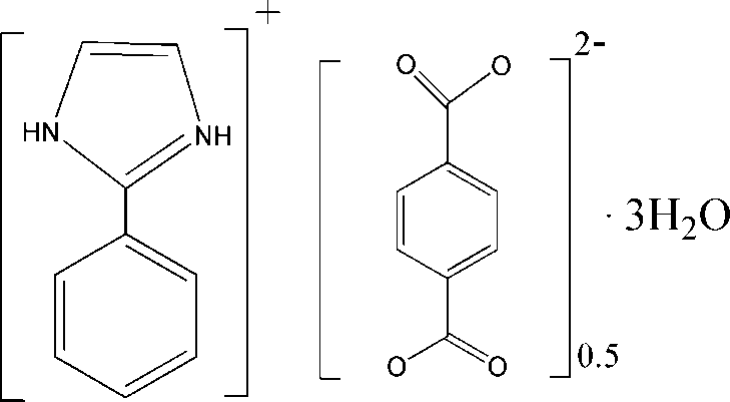


## Experimental

### 

#### Crystal data

C_9_H_9_N_2_^+^·0.5C_8_H_4_O_4_^2−^·3H_2_O
*M* *_r_* = 281.29
Triclinic, 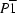

*a* = 7.208 (1) Å
*b* = 9.164 (2) Å
*c* = 11.368 (2) Å
α = 78.506 (1)°
β = 75.478 (5)°
γ = 86.774 (5)°
*V* = 712.3 (2) Å^3^
*Z* = 2
Mo *K*α radiation
μ = 0.10 mm^−1^
*T* = 293 K
0.17 × 0.15 × 0.12 mm

#### Data collection

Bruker APEX diffractometer
Absorption correction: multi-scan (*SADABS*; Sheldrick, 1996 ▶) *T*_min_ = 0.55, *T*_max_ = 0.72
4505 measured reflections
2611 independent reflections
1519 reflections with *I* > 2σ(*I*)
*R*_int_ = 0.044

#### Refinement

*R*[*F* ^2^ > 2σ(*F* ^2^)] = 0.051
*wR*(*F* ^2^) = 0.120
*S* = 0.99
2611 reflections
205 parameters
9 restraints
H atoms treated by a mixture of independent and constrained refinement
Δρ_max_ = 0.18 e Å^−3^
Δρ_min_ = −0.22 e Å^−3^

### 

Data collection: *SMART* (Bruker, 1997 ▶); cell refinement: *SAINT* (Bruker, 1997 ▶); data reduction: *SAINT*; program(s) used to solve structure: *SHELXS97* (Sheldrick, 2008 ▶); program(s) used to refine structure: *SHELXL97* (Sheldrick, 2008 ▶); molecular graphics: *SHELXTL* (Sheldrick, 2008 ▶); software used to prepare material for publication: *SHELXTL*.

## Supplementary Material

Crystal structure: contains datablock(s) global, I. DOI: 10.1107/S1600536811044953/bt5686sup1.cif

Structure factors: contains datablock(s) I. DOI: 10.1107/S1600536811044953/bt5686Isup2.hkl

Additional supplementary materials:  crystallographic information; 3D view; checkCIF report

## Figures and Tables

**Table 1:** Hydrogen-bond geometry (Å, °)

*D*—H⋯*A*	*D*—H	H⋯*A*	*D*⋯*A*	*D*—H⋯*A*
N1—H1*A*⋯O1	0.86	1.91	2.768 (3)	172
N2—H2*A*⋯O3*W*^i^	0.86	1.82	2.663 (3)	168
O1*W*—H*W*11⋯O2	0.85 (1)	2.02 (1)	2.850 (3)	167 (3)
O1*W*—H*W*12⋯O2^ii^	0.85 (1)	2.33 (3)	2.955 (3)	131 (3)
O2*W*—H*W*21⋯O1^iii^	0.86 (1)	2.03 (2)	2.818 (3)	153 (3)
O2*W*—H*W*22⋯O2^ii^	0.85 (1)	2.01 (1)	2.828 (3)	161 (3)
O3*W*—H*W*31⋯O1*W*^iv^	0.85 (1)	1.94 (2)	2.749 (4)	160 (4)
O3*W*—H*W*32⋯O2*W*^v^	0.85 (1)	1.89 (1)	2.723 (4)	167 (4)

Symmetry codes: (i) 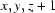; (ii) 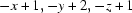; (iii) 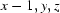; (iv) 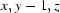; (v) 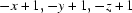.

**Fig. 1.**
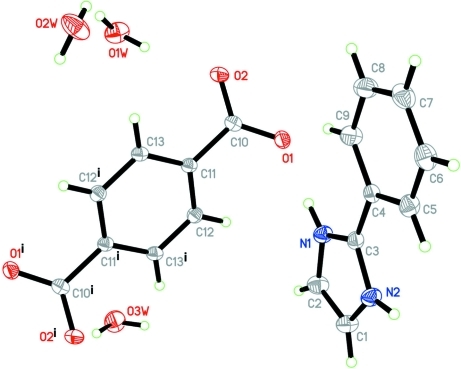
The structure of the title compound showing the atomic numbering scheme. Displacement ellipsoids are drawn at the 30% probability level. Symmetry codes: (i) 1 - x, 1 - y, 1 - z.

## supplementary crystallographic information

### Comment

2-Phenylimidazolium nitrate and 2-phenylimidazolium acetate have been reported (Xia *et al.*, 2009; Xia & Yao, 2010). Here, I report the synthesis and crystal structure of the 2-phenylimidazolium hemi-benzene-1,4-dicarboxylate trihydrate.

The asymmetric unit of the title compound is composed of one 2-phenylimidazolium cation, half a benzene-1,4-dicarboxylate anion, and three water molecules (Fig. 1) which are connected by O—H···O and N—H···O hydrogen bonds to a three-dimensional network.

### Experimental

A mixture of 2-phenylimidazole (0.5 mmol), benzene-1,4-dicarboxylic acid (0.3 mmol) and H_2_O (10 ml) was mixed. After several days, colorless crystals were obtained at room temperature.

### Refinement

All H atoms on C and N atoms were positioned geometrically (N—H = 0.86 Å and C—H = 0.93 Å) and refined as riding, with *U*_iso_(H)=1.2*U*_eq_(carrier). Water H atoms were located in a difference Fourier map and refined as riding with the O—H and H···H distances restrainted to 0.85±0.01 and 1.35±0.01 Å, respectively.

### Crystal data

**Table:**

C_9_H_9_N_2_^+^·0.5C_8_H_4_O_4_^2^^−^·3H_2_O	*Z* = 2
*M**_r_* = 281.29	*F*(000) = 298
Triclinic, *P*1	*D*_x_ = 1.311 Mg m^−^^3^
Hall symbol: -P 1	Mo *K*α radiation, λ = 0.71073 Å
*a* = 7.208 (1) Å	Cell parameters from 4505 reflections
*b* = 9.164 (2) Å	θ = 1.9–25.4°
*c* = 11.368 (2) Å	µ = 0.10 mm^−^^1^
α = 78.506 (1)°	*T* = 293 K
β = 75.478 (5)°	Block, colorless
γ = 86.774 (5)°	0.17 × 0.15 × 0.12 mm
*V* = 712.3 (2) Å^3^	

### Data collection

**Table:**

Bruker APEX diffractometer	2611 independent reflections
Radiation source: fine-focus sealed tube	1519 reflections with *I* > 2σ(*I*)
graphite	*R*_int_ = 0.044
φ and ω scans	θ_max_ = 25.4°, θ_min_ = 1.9°
Absorption correction: multi-scan (*SADABS*; Sheldrick, 1996)	*h* = −8→7
*T*_min_ = 0.55, *T*_max_ = 0.72	*k* = −10→11
4505 measured reflections	*l* = −13→11

### Refinement

**Table:**

Refinement on *F*^2^	Primary atom site location: structure-invariant direct methods
Least-squares matrix: full	Secondary atom site location: difference Fourier map
*R*[*F*^2^ > 2σ(*F*^2^)] = 0.051	Hydrogen site location: inferred from neighbouring sites
*wR*(*F*^2^) = 0.120	H atoms treated by a mixture of independent and constrained refinement
*S* = 0.99	*w* = 1/[σ^2^(*F*_o_^2^) + (0.0385*P*)^2^] where *P* = (*F*_o_^2^ + 2*F*_c_^2^)/3
2611 reflections	(Δ/σ)_max_ < 0.001
205 parameters	Δρ_max_ = 0.18 e Å^−^^3^
9 restraints	Δρ_min_ = −0.22 e Å^−^^3^

### Special details

**Table:**

Geometry. All e.s.d.'s (except the e.s.d. in the dihedral angle between two l.s. planes) are estimated using the full covariance matrix. The cell e.s.d.'s are taken into account individually in the estimation of e.s.d.'s in distances, angles and torsion angles; correlations between e.s.d.'s in cell parameters are only used when they are defined by crystal symmetry. An approximate (isotropic) treatment of cell e.s.d.'s is used for estimating e.s.d.'s involving l.s. planes.
Refinement. Refinement of *F*^2^ against ALL reflections. The weighted *R*-factor *wR* and goodness of fit *S* are based on *F*^2^, conventional *R*-factors *R* are based on *F*, with *F* set to zero for negative *F*^2^. The threshold expression of *F*^2^ > σ(*F*^2^) is used only for calculating *R*-factors(gt) *etc*. and is not relevant to the choice of reflections for refinement. *R*-factors based on *F*^2^ are statistically about twice as large as those based on *F*, and *R*- factors based on ALL data will be even larger.

### Fractional atomic coordinates and isotropic or equivalent isotropic displacement parameters (Å^2^)

**Table:**

	*x*	*y*	*z*	*U*_iso_*/*U*_eq_	
C1	0.9035 (4)	0.2035 (3)	0.8466 (3)	0.0553 (7)	
H1	0.9328	0.1023	0.8563	0.066*	
C2	0.9084 (4)	0.2981 (3)	0.7392 (3)	0.0543 (7)	
H2	0.9410	0.2750	0.6605	0.065*	
C3	0.8178 (3)	0.4256 (2)	0.8904 (2)	0.0391 (6)	
C4	0.7537 (3)	0.5458 (2)	0.9583 (2)	0.0386 (6)	
C5	0.7153 (4)	0.5189 (3)	1.0870 (2)	0.0514 (7)	
H5	0.7339	0.4241	1.1303	0.062*	
C6	0.6498 (4)	0.6324 (3)	1.1502 (3)	0.0604 (8)	
H6	0.6230	0.6135	1.2362	0.072*	
C7	0.6238 (4)	0.7730 (3)	1.0875 (3)	0.0641 (9)	
H7	0.5806	0.8495	1.1306	0.077*	
C8	0.6617 (4)	0.8005 (3)	0.9604 (3)	0.0639 (8)	
H8	0.6437	0.8958	0.9177	0.077*	
C9	0.7263 (4)	0.6880 (3)	0.8957 (3)	0.0542 (7)	
H9	0.7515	0.7076	0.8097	0.065*	
C10	0.7373 (3)	0.7275 (2)	0.5307 (2)	0.0387 (6)	
C11	0.6137 (3)	0.6093 (2)	0.5159 (2)	0.0342 (6)	
C12	0.6925 (3)	0.4735 (2)	0.4926 (2)	0.0381 (6)	
H12	0.8223	0.4555	0.4873	0.046*	
C13	0.4193 (3)	0.6353 (2)	0.5229 (2)	0.0373 (6)	
H13	0.3647	0.7262	0.5380	0.045*	
N1	0.8561 (3)	0.4352 (2)	0.76772 (18)	0.0446 (5)	
H1A	0.8490	0.5154	0.7148	0.054*	
N2	0.8474 (3)	0.2839 (2)	0.93913 (19)	0.0483 (6)	
H2A	0.8333	0.2485	1.0168	0.058*	
O1	0.8703 (2)	0.68667 (16)	0.58457 (15)	0.0455 (5)	
O2	0.7039 (3)	0.86002 (17)	0.48727 (17)	0.0574 (5)	
O1W	0.4795 (4)	0.9746 (2)	0.31515 (19)	0.0722 (6)	
O2W	0.0067 (4)	0.9337 (3)	0.6501 (2)	0.0830 (7)	
O3W	0.7934 (5)	0.1375 (3)	0.1721 (2)	0.0883 (7)	
HW11	0.529 (4)	0.934 (4)	0.374 (2)	0.127 (16)*	
HW12	0.373 (3)	1.010 (4)	0.350 (3)	0.132 (17)*	
HW21	0.004 (4)	0.858 (2)	0.616 (3)	0.106 (13)*	
HW22	0.084 (4)	0.994 (2)	0.595 (2)	0.109 (14)*	
HW31	0.683 (2)	0.107 (4)	0.214 (3)	0.132 (18)*	
HW32	0.862 (4)	0.129 (4)	0.224 (3)	0.120 (15)*	

### Atomic displacement parameters (Å^2^)

**Table:**

	*U*^11^	*U*^22^	*U*^33^	*U*^12^	*U*^13^	*U*^23^
C1	0.0659 (19)	0.0421 (14)	0.0546 (19)	0.0025 (14)	−0.0106 (15)	−0.0078 (14)
C2	0.0643 (19)	0.0479 (16)	0.0483 (18)	0.0019 (14)	−0.0064 (14)	−0.0133 (14)
C3	0.0400 (14)	0.0422 (14)	0.0330 (15)	−0.0070 (11)	−0.0096 (11)	0.0002 (11)
C4	0.0368 (14)	0.0431 (14)	0.0368 (15)	−0.0047 (11)	−0.0102 (11)	−0.0075 (11)
C5	0.0567 (17)	0.0583 (16)	0.0380 (17)	−0.0048 (13)	−0.0112 (13)	−0.0061 (13)
C6	0.0636 (19)	0.077 (2)	0.0435 (18)	−0.0051 (16)	−0.0107 (15)	−0.0196 (16)
C7	0.063 (2)	0.071 (2)	0.067 (2)	0.0036 (16)	−0.0152 (17)	−0.0362 (18)
C8	0.080 (2)	0.0494 (17)	0.065 (2)	0.0021 (16)	−0.0202 (18)	−0.0141 (15)
C9	0.0691 (19)	0.0492 (16)	0.0425 (17)	−0.0030 (14)	−0.0094 (14)	−0.0092 (13)
C10	0.0456 (16)	0.0362 (13)	0.0327 (15)	−0.0064 (11)	−0.0046 (12)	−0.0076 (11)
C11	0.0417 (14)	0.0314 (12)	0.0288 (14)	−0.0005 (11)	−0.0085 (11)	−0.0045 (10)
C12	0.0383 (14)	0.0390 (13)	0.0363 (15)	0.0003 (11)	−0.0090 (11)	−0.0056 (11)
C13	0.0431 (15)	0.0300 (12)	0.0389 (15)	0.0019 (11)	−0.0090 (11)	−0.0083 (10)
N1	0.0526 (13)	0.0421 (12)	0.0362 (13)	−0.0008 (10)	−0.0090 (10)	−0.0027 (9)
N2	0.0594 (14)	0.0436 (12)	0.0375 (13)	−0.0023 (11)	−0.0102 (11)	0.0011 (10)
O1	0.0495 (11)	0.0452 (9)	0.0447 (11)	−0.0091 (8)	−0.0194 (9)	−0.0032 (8)
O2	0.0735 (13)	0.0318 (9)	0.0722 (14)	−0.0085 (9)	−0.0346 (11)	0.0010 (9)
O1W	0.0924 (18)	0.0683 (14)	0.0497 (14)	0.0082 (13)	−0.0121 (13)	−0.0059 (11)
O2W	0.127 (2)	0.0710 (14)	0.0531 (15)	−0.0447 (15)	−0.0272 (14)	0.0016 (12)
O3W	0.106 (2)	0.0951 (18)	0.0560 (15)	−0.0288 (16)	−0.0322 (17)	0.0270 (13)

### Geometric parameters (Å, °)

**Table:**

C1—C2	1.343 (3)	C9—H9	0.9300
C1—N2	1.369 (3)	C10—O2	1.251 (3)
C1—H1	0.9300	C10—O1	1.263 (3)
C2—N1	1.368 (3)	C10—C11	1.501 (3)
C2—H2	0.9300	C11—C12	1.385 (3)
C3—N2	1.334 (3)	C11—C13	1.393 (3)
C3—N1	1.338 (3)	C12—C13^i^	1.381 (3)
C3—C4	1.458 (3)	C12—H12	0.9300
C4—C9	1.384 (3)	C13—C12^i^	1.381 (3)
C4—C5	1.393 (3)	C13—H13	0.9300
C5—C6	1.376 (3)	N1—H1A	0.8600
C5—H5	0.9300	N2—H2A	0.8600
C6—C7	1.371 (4)	O1W—HW11	0.852 (10)
C6—H6	0.9300	O1W—HW12	0.850 (10)
C7—C8	1.375 (4)	O2W—HW21	0.859 (10)
C7—H7	0.9300	O2W—HW22	0.854 (10)
C8—C9	1.377 (3)	O3W—HW31	0.850 (10)
C8—H8	0.9300	O3W—HW32	0.847 (10)
			
C2—C1—N2	107.1 (2)	C8—C9—C4	120.2 (3)
C2—C1—H1	126.4	C8—C9—H9	119.9
N2—C1—H1	126.4	C4—C9—H9	119.9
C1—C2—N1	106.9 (2)	O2—C10—O1	124.1 (2)
C1—C2—H2	126.6	O2—C10—C11	117.9 (2)
N1—C2—H2	126.6	O1—C10—C11	118.0 (2)
N2—C3—N1	106.71 (19)	C12—C11—C13	119.0 (2)
N2—C3—C4	126.4 (2)	C12—C11—C10	120.3 (2)
N1—C3—C4	126.8 (2)	C13—C11—C10	120.69 (19)
C9—C4—C5	119.0 (2)	C13^i^—C12—C11	120.8 (2)
C9—C4—C3	120.4 (2)	C13^i^—C12—H12	119.6
C5—C4—C3	120.6 (2)	C11—C12—H12	119.6
C6—C5—C4	120.1 (2)	C12^i^—C13—C11	120.2 (2)
C6—C5—H5	120.0	C12^i^—C13—H13	119.9
C4—C5—H5	120.0	C11—C13—H13	119.9
C7—C6—C5	120.6 (3)	C3—N1—C2	109.7 (2)
C7—C6—H6	119.7	C3—N1—H1A	125.2
C5—C6—H6	119.7	C2—N1—H1A	125.2
C6—C7—C8	119.7 (2)	C3—N2—C1	109.6 (2)
C6—C7—H7	120.2	C3—N2—H2A	125.2
C8—C7—H7	120.2	C1—N2—H2A	125.2
C7—C8—C9	120.5 (3)	HW11—O1W—HW12	104.8 (15)
C7—C8—H8	119.7	HW21—O2W—HW22	103.8 (14)
C9—C8—H8	119.7	HW31—O3W—HW32	106.0 (16)

Symmetry codes: (i) −*x*+1, −*y*+1, −*z*+1.

### Hydrogen-bond geometry (Å, °)

**Table:**

*D*—H···*A*	*D*—H	H···*A*	*D*···*A*	*D*—H···*A*
N1—H1A···O1	0.86	1.91	2.768 (3)	172.
N2—H2A···O3W^ii^	0.86	1.82	2.663 (3)	168.
O1W—HW11···O2	0.85 (1)	2.02 (1)	2.850 (3)	167 (3)
O1W—HW12···O2^iii^	0.85 (1)	2.33 (3)	2.955 (3)	131 (3)
O2W—HW21···O1^iv^	0.86 (1)	2.03 (2)	2.818 (3)	153 (3)
O2W—HW22···O2^iii^	0.85 (1)	2.01 (1)	2.828 (3)	161 (3)
O3W—HW31···O1W^v^	0.85 (1)	1.94 (2)	2.749 (4)	160 (4)
O3W—HW32···O2W^i^	0.85 (1)	1.89 (1)	2.723 (4)	167 (4)

Symmetry codes: (ii) *x*, *y*, *z*+1; (iii) −*x*+1, −*y*+2, −*z*+1; (iv) *x*−1, *y*, *z*; (v) *x*, *y*−1, *z*; (i) −*x*+1, −*y*+1, −*z*+1.

## References

[bb1] Bruker (1997). *SMART* and *SAINT* Bruker AXS Inc., Madison, Wisconsin, USA.

[bb2] Sheldrick, G. M. (1996). *SADABS* University of Göttingen, Germany.

[bb3] Sheldrick, G. M. (2008). *Acta Cryst.* A**64**, 112–122. 10.1107/S0108767307043930 18156677

[bb4] Xia, D.-C., Li, W.-C. & Han, S. (2009). *Acta Cryst.* E**65**, o3283. 10.1107/S1600536809050703 PMC2972149 21578976

[bb5] Xia, D.-C. & Yao, J.-H. (2010). *Acta Cryst.* E**66**, o649. 10.1107/S1600536810004939 PMC2983526 21580402

